# Early Hospital Arrival After Acute Ischemic Stroke Is Associated With Family Members' Knowledge About Stroke

**DOI:** 10.3389/fneur.2021.652321

**Published:** 2021-05-26

**Authors:** Rongyu Wang, Zhiqiang Wang, Dongdong Yang, Jian Wang, Chongji Gou, Yaodan Zhang, Liulin Xian, Qingsong Wang

**Affiliations:** ^1^Department of Neurology, Hospital of Chengdu University of Traditional Chinese Medicine, Chengdu, China; ^2^Department of Neurology, Yaan People's Hospital, Yaan, China; ^3^Department of Neurology, Pengzhou People's Hospital, Pengzhou, China; ^4^Department of Neurology, The Affiliated Hospital of North Sichuan Medical College, Nanchong, China; ^5^Department of Neurology, Nanbu Traditional Chinese Medicine, Nanbu, China; ^6^Department of Neurology, The General Hospital of Western Theater Command, Chengdu, China

**Keywords:** acute ischemic stroke, prehospital delay, prevalence, family member, independent factors

## Abstract

**Background and Purpose:** Prehospital delay is the major factor limiting intravenous thrombolysis and mechanical thrombectomy in acute ischemic stroke (AIS). This study aimed to: (1) identify factors related to prehospital delay and (2) determine the impact of recognition and behavior of family members on patient delay.

**Methods:** A cross-sectional, multicenter study was conducted at six teaching hospitals in China between December 1, 2018 and November 30, 2019. Patients who experienced AIS within 7 days of onset were interviewed.

**Results:** Of 1,782 consecutive patients (male, 57.97%; mean age, 66.3 ± 9.65 years) who had an AIS, 267 (14.98%) patients arrived within 4.5 h and 722 (40.52%) patients arrived within 6 h of stroke onset. Among patients who arrived within 4.5 h, 103 (38.6%) received thrombolysis. Age over 65 years (OR, 2.009; 95% CI, 1.014–3.982), prior stroke (OR, 3.478; 95% CI, 1.311–9.229), blurred vision (OR, 3.95; 95% CI, 1.71–9.123), and patients deciding to seek medical help (OR, 3.097; 95% CI, 1.417–6.769) were independently associated with late arrival. In contrast, sudden onset of symptoms (OR, 0.075; 95% CI, 0.028–0.196), the National Institutes of Health Stroke Scale 7–15 (OR, 0.093; 95% CI, 0.035–0.251), consciousness disturbance (OR, 0.258; 95% CI, 0.091–0.734), weakness (OR, 0.265; 95% CI, 0.09–0.784), arrival by ambulance (OR, 0.102; 95% CI, 0.049–0.211), decision time <30 min (OR, 0.008; 95% CI, 0.003–0.018), and family member understanding stroke requires early treatment (OR, 0.224; 95% CI, 0.109–0.462) were independently associated with early arrival.

**Conclusions:** The prehospital delay in China lags behind Western countries. Recognition and behavior of stroke patients' family members may play a key role in early arrival.

## Introduction

Delayed hospital arrival has been identified as the most significant prehospital barrier to thrombolysis within 4.5 h or to mechanical thrombectomy within 6 h for acute ischemic stroke (AIS) (1). The problem of treatment delays is global, but the factors causing prolonged prehospital delays may be different due to variations in ethnicity, culture, socioeconomic features, and health care systems (2, 3). Existed findings suggest that bystander's quick response plays a key role in shortening delays when the stroke occurs. The bystander's timely recognition, action, and emotional response are all associated with hospital arrival within the thrombolysis time window (4, 5). Influenced by traditional Chinese culture, a majority of the elderly would live with their descendants, which has been confirmed to happen much more frequently in China (6–8) than in other countries (9–11). Patients, whose symptoms were discovered by family rather than themselves arrived at the hospital earlier than those who do not live with family (8, 12). Therefore, it is necessary to investigate how factors of family members' cognition and behaviors could influence the prehospital delay.

## Methods

### Study Setting

This was a cross-sectional multi-center study that included consecutive hospitalized AIS patients to evaluate the relation between cognition and behaviors of patients' family members and prehospital delays. The patients, along with their family members, were recruited from six teaching hospitals in China between December 1, 2018, and November 30, 2019. The criteria for the participating patients were as follows: (1) age ≥18 years; (2) AIS was diagnosed by clinical examination and confirmed by brain CT or MRI scan within 7 days of symptom onset. Exclusion criteria included: (1) patients whose symptoms onset at hospitals or nursing homes where there were professional medical personnel; (2) patients or their family members who refused to participate in this investigation or were unable to complete the study interview; and (3) patients who were unable to define the symptom onset time since they did not notice their symptoms until they got up.

### Data Collection

The questionnaire was designed based on reviewing guidelines (13) and similar previous studies (7, 14). Variables evaluated in this questionnaire included sociodemographic factors (age, sex, marital status, live status, etc.), socioeconomic factors (monthly income, medical insurance), medical history factors (stroke history, hypertension, diabetes, hyperlipidemia, atrial fibrillation, drinking, and smoking), onset circumstances and symptoms, the score of National Institutes of Health Stroke Scale (NIHSS), arriving by referral or ambulance, and both patients and their family members' cognitive and behavioral factors. For cognitive and behavioral factors, the data of knowledge about stroke (stroke education, the ability to describe FAST and recognize the stroke, understood the time window and the early treatment of stroke), and initial reactions of patients and their family members would be assessed. The Data were collected within 48 h of hospital admission, with a structured interview conducted by a trained neurologist. When patients encountered speech difficulties or a consciousness disturbance, their caretakers or family members were interviewed. The medical records for patients with AIS diagnosis would also be collected.

### Definition

The time of symptom onset was defined as the time when stroke-related symptoms first occurred. The prehospital delay was defined as the period from symptom onset to the earliest documented time in the emergency department or the general department of participating hospitals. The prehospital delay was divided into 4.5 or more hours and 6 or more hours of delay. Decision time was defined as the period from symptoms onset to reaching out for medical services. The decision-maker was defined as the person who deemed medical attention necessary and sought medical help. The patient's family members represented the caregiver or the decision-maker who sought medical help for the patient. The monthly income was divided into four levels (<1,000, 1,000–3,000, 3,000–5,000, and >5,000 Yuan RMB) based on the overall income standards in Western China; the living status was divided into living alone, living with descendants, or living in the apartment for the aged; the living place was divided into living in an urban area, a suburban area, or a rural area; the education level was divided into <6 years, 6–12 years, or longer than 12 years; the driving time was divided into <30 min, 30–60 min, or over 60 min; the place of symptom onset was divided into at home or in other places; and the first reaction was divided into calling an ambulance, going to a hospital directly, and contacting and waiting for relative. Onset symptoms of patient's complaint included: unconsciousness, weakness (limbs or one side of the body), numbness (face or limbs), abnormal gait, speech difficulty, blurred vision (unclear vision in one or both eyes), and dizziness.

### Ethics

This study was approved by the Research Ethics Committee of Chengdu Military General Hospital, Chengdu, China. All of the study subjects read the purpose statement of the investigation and each provided written informed consent. The study was conducted in accordance with the Declaration of Helsinki, and the privacy of the patients was strictly protected.

### Statistical Analysis

Descriptive statistics: mean [± standard deviation (SD)] and median [interquartile range (IQR)]. All analyses were conducted using Statistical Product and Service Solutions (SPSS) software. Time to presentation was divided into two groups: the early arrival group (within 4.5 or 6 h of symptom onset) and the delayed arrival group (≥4.5 or 6 h of symptom onset). A χ^2^ test was used to evaluate the significance of associations. Next, forward stepwise logistic regression analysis was performed to identify the independent predictors of prehospital delays and thrombolysis. Criteria for the entry and removal of variables in the regression analyses were set at *P* < 0.05 and *P* > 0.10, respectively. Statistical significance was accepted as *P* < 0.05.

## Results

### Participants' Baseline Characteristics

**Figure 1:**
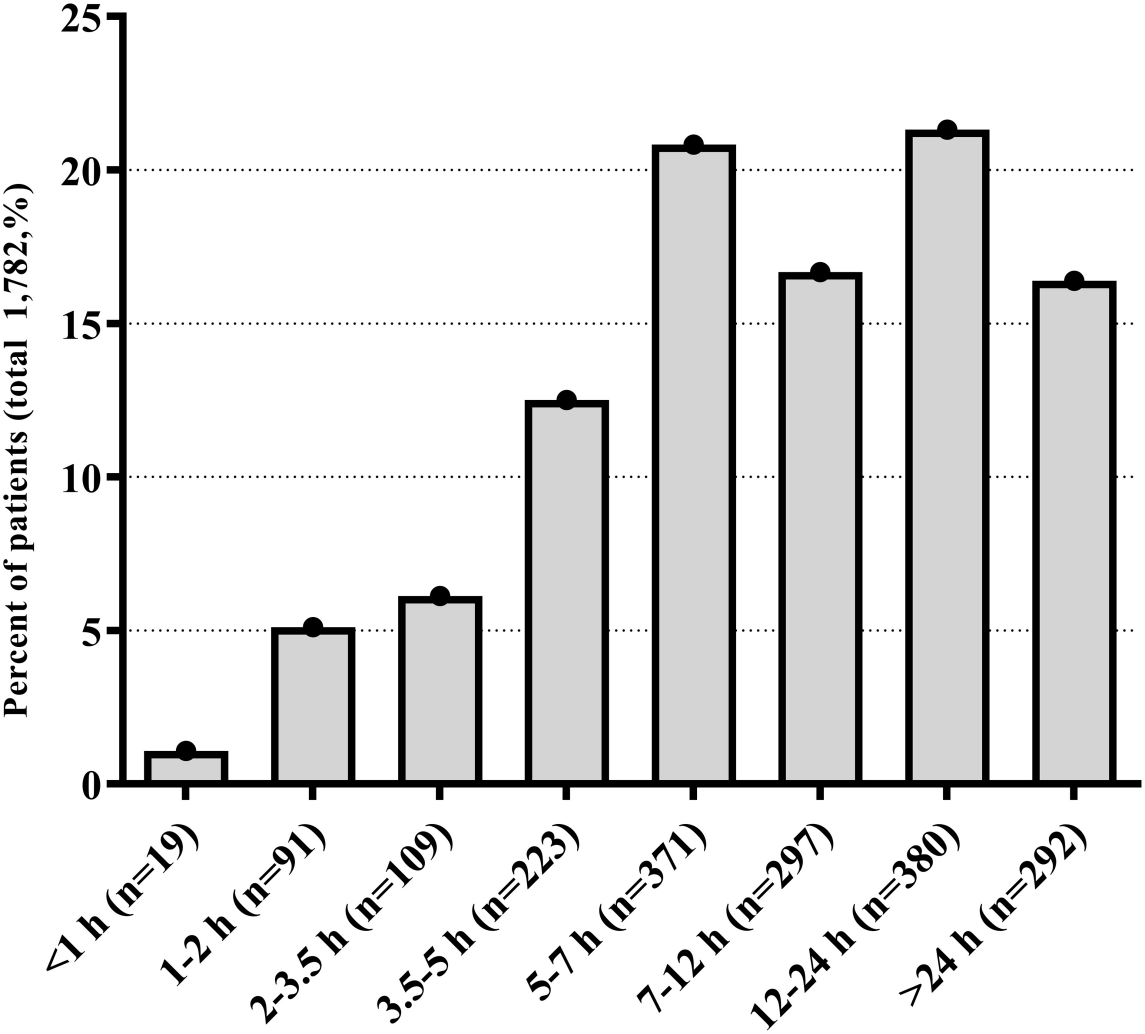
Frequency distribution of prehospital delay time for patients with acute stroke.

Of the 1,985 patients who were initially screened for eligibility, 203 (10.23%) cases were excluded for the following reasons: 161 (8.11%) patients or their relatives refused to participate, 11 (0.55%) patients had symptom onset at hospitals or nursing homes, and 31 (1.56%) patients did not provide complete information. Finally, a total of 1,782 patients (male, 57.97%; mean age, 66.3±9.65 years) were enrolled in our analysis. The median National Institutes of Health Stroke Scale (NIHSS) score was 10 (IQR = 6–14). The frequency distribution of prehospital delay for patients with AIS is shown in the Figure 1. The median prehospital delay of all subjects was 410 min (IQR = 62–1,140). Among these patients, 103 (5.8%) of them received intravenous thrombolysis therapy, 267 (14.98%) arrived at hospitals within 4.5 h, and 722 (40.52%) arrived within 6 h of the symptom onset. Most patients lived with their descendants, and the most common onset symptom was weakness (72.9%) and numbness (59.9%). In terms of their first reaction, 390 (21.9%) patients' initial reaction was to call an ambulance, 591 (33.2%) patients went directly to a hospital, and 26.1% preferred to contact relatives. Results showed that only 286 (16.0%) patients were delivered to the hospital by an ambulance, and the mean time from they called the ambulance until they arrived at the hospital was 103 ± 52.6 min. More detailed information and univariate analysis of factors related to prehospital delay based on arrival time are presented in Supplementary Table 1. Most results of the 6-h delay were consistent with that of the 4.5-h delay.

### Independent Factors Associated With Prehospital Delay (4.5 h)

Multivariable logistic regression analysis (Table 1) identified the factors that were independently associated with prehospital delay (4.5 h). Subjects who were 65 years old or above [odds ratio (OR), 2.009; 95% confidence interval (CI), 1.014–3.982], had a prior stroke or transient ischemic attack (OR, 3.478; 95% CI, 1.311–9.229), or had blurred vision (OR, 3.95; 95% CI, 1.71–9.123), and made the decision of seeking medical services by themselves (OR, 3.097; 95% CI, 1.417–6.769) were independently associated with late arrival. In contrast, those who encountered sudden symptom onset (OR, 0.075; 95% CI, 0.028–0.196), NIHSS 7–15 (OR, 0.093; 95% CI, 0.035–0.251), or >15 (OR, 0.04; 95% CI, 0.013–0.13), consciousness disturbance (OR, 0.258; 95% CI, 0.091–0.734) or weakness (OR, 0.265; 95% CI, 0.09–0.784), arrived by an ambulance (OR, 0.102; 95% CI, 0.049–0.211), made decision in <30 min (OR, 0.008; 95% CI, 0.003–0.018) or 30–60 min (OR, 0.003; 95% CI, 0.001–0.008), and had family members who knew early treatment for stroke (OR, 0.224; 95% CI, 0.109–0.462) were independently associated with early arrival.

**Table 1:** Logistic regression model of potential determinants for delay (4.5 h).

**Factors**	**Total (%)**	**>4.5 h (%)**	***P***	**OR**	**95% C.I**.
Age ≥ 65 years	1,186 (66.6%)	1,053 (88.8%)	0.046	2.009	(1.014–3.982)
Prior stroke	397 (22.3%)	376 (94.7%)	0.012	3.478	(1.311–9.229)
Symptom onset sudden	1,287 (72.2%)	1,039 (80.7%)	<0.001	0.075	(0.028–0.196)
**NIHSS**					
<7	517 (29.0%)	501 (96.9%)		1	
7–15	946 (53.1%)	776 (82.0%)	<0.001	0.093	(0.035–0.251)
>15	319 (17.9%)	238 (74.6%)	<0.001	0.04	(0.013–0.13)
Disturbance of consciousness	110 (6.2%)	57 (51.8%)	0.011	0.258	(0.091–0.734)
Weakness	1,299 (72.9%)	1,046 (80.5%)	0.016	0.265	(0.09–0.784)
Blurred version	523 (29.3%)	415 (79.3%)	0.001	3.95	(1.71–9.123)
Arrived by ambulance	286 (16.1%)	95 (33.2%)	<0.001	0.102	(0.049–0.211)
Patient decided to seek medical services	1,001 (56.2%)	912 (91.1%)	0.005	3.097	(1.417–6.769)
**Decision time**					
>60 min	1,438 (80.7%)	1,424 (99.0%)		1	
30–60 min	164 (9.2%)	63 (38.8%)	<0.001	0.003	(0.001–0.008)
<30 min	180 (10.1%)	28 (15.6%)	<0.001	0.008	(0.003–0.018)
Understand that stroke requires early treatment (family)	670 (37.6%)	462 (69.0%)	<0.001	0.224	(0.109–0.462)
**Education (family)**					
<6 years	263 (14.8%)	256 (97.3%)		1	
6–12 years	902 (50.6%)	817 (90.6%)	0.969	0.968	
>12 years	617 (34.6%)	442 (71.6%)	0.111	0.266	

*N, number; OR, odds ratio; CI, confidence interval. Weakness, blurred vision: the patients' complaint*.

### Independent Factors Associated With Prehospital Delay (6 h)

Results of the final multilevel logistic regression model for the 6-h cutoff point are shown in Table 2. For subjects who were 65 years old or above (OR, 1.392; 95% CI, 1.026–1.89), lived in suburban (OR, 2.55; 95% CI, 1.875–3.467) or rural areas (OR, 7.399; 95% CI, 4.26–12.644), had a driving time of 30–60 min (OR, 2.011; 95% CI, 1.28–3.159) or over 60 min (OR, 1.766; 95% CI, 1.195–2.61), had a prior stroke (OR, 4.246; 95% CI, 2.85–6.326), had a history of hyperlipidemia (OR, 2.096; 95% CI, 1.291–3.402) or atrial fibrillation (OR, 2.078; 95% CI, 1.199–3.603), had a gait disturbance(OR, 1.97; 95% CI, 1.22–3.182), initially reacted to symptoms by going directly to the hospital compared with by ambulance (OR, 2.37; 95% CI, 1.493–3.763), contacting relatives (OR, 2.413; 95% CI, 1.478–3.94), or waiting for symptoms to disappear (OR, 1.954; 95% CI, 1.187–3.215), transferred from another hospital(OR, 5.725; 95% CI, 3.151–10.399), and made the decisions to seek medical services by themselves (OR, 2.346; 95% CI, 1.484–3.708) were independently associated with late arrival. In contrast, for these who lived with their descendants (OR, 0.07; 95% CI, 0.019–0.253), onset at daytime (OR, 0.393; 95% CI, 0.257–0.602), sudden onset of symptoms (OR, 0.321; 95% CI, 0.227–0.453), arrived by ambulance (OR, 0.266; 95% CI, 0.14–0.503), patients have received stroke education (OR, 0.423; 95% CI, 0.288–0.622), patients' ability to describe FAST(Face, Arm, Speech, Time) (OR, 0.48; 95% CI, 0.303–0.759), patients recognized the problem as a stroke (OR, 0.165; 95% CI, 0.098–0.278), decision time <60 min [30–60 min: (OR, 0.036; 95% CI, 0.013–0.103), or <30 min: (OR, 0.012; 95% CI, 0.004–0.036)], and family members knew that strokes require early treatment (OR, 0.542; 95% CI, 0.398–0.739) were independently associated with early arrival.

**Table 2:** Logistic regression model of potential determinants for delay (6 h).

**Factors**	**Total**	**>6 h (%)**	***P***	**OR**	**95% CI**
Age ≥ 65 years	1,186 (66.5%)	756 (63.7%)	0.034	1.392	(1.026–1.89)
**Lives**					
Alone	71 (4.0%)	66 (93.0%)		1	
With descendants	1,692 (95.0%)	981 (58.0%)	<0.001	0.07	(0.019–0.253)
Senile apartments	19 (1.0%)	13 (68.4%)	0.338	0.316	
**Resides in**					
Urban	928 (52.1%)	404 (43.5%)		1	
Suburban	577 (32.4%)	426 (73.8%)	<0.001	2.55	(1.875–3.467)
Rural	277 (15.5%)	230 (83.0%)	<0.001	7.339	(4.26–12.644)
**Driving time**					
<30 min	327 (18.4%)	155 (47.4%)		1	
30–60 min	493 (27.7%)	272 (55.2%)	0.002	2.011	(1.28–3.159)
>60 min	962 (54.0%)	633 (65.8%)	0.004	1.766	(1.195–2.61)
Prior stroke	397 (22.3%)	322 (53.3%)	<0.001	4.246	(2.85–6.326)
Sudden onset	1,287 (72.2%)	697 (54.2%)	<0.001	0.321	(0.227–0.453)
History of hyperlipidemia	233 (13.1%)	151 (64.8%)	0.003	2.096	(1.291–3.402)
History of atrial fibrillation	134 (7.5%)	100 (74.6%)	0.009	2.078	(1.199–3.603)
Daytime onset	1,488 (83.5%)	832 (55.9%)	<0.001	0.393	(0.257–0.602)
Gait disturbance	663 (37.2%)	345 (52.0%)	0.006	1.97	(1.22–3.182)
Arrived by ambulance	286 (16.0%)	26 (9.1%)	<0.001	0.266	(0.14–0.503)
Patient decided to seek medical service	1,029 (57.7%)	691 (67.2%)	<0.001	2.346	(1.484–3.708)
**Decision time**					
>60 min	1,438 (80.7%)	1,046 (72.7%)		1	
30–60 min	164 (9.2%)	6 (3.7%)	<0.001	0.036	(0.013–0.103)
<30 min	180 (10.1%)	8 (4.4%)	<0.001	0.012	(0.004–0.036)
**Patient's initial reaction**					
Called 120	390 (21.9%)	91 (23.3%)		1	
Went directly to hospital	591 (33.2%)	426 (72.1%)	<0.001	2.37	(1.493–3.763)
Contacted relative/acquaintance	465 (26.1%)	321 (69.0%)	<0.001	2.413	(1.478–3.94)
Waited for symptoms to go away	336 (18.8%)	222 (66.1%)	0.008	1.954	(1.187–3.215)
Arrival through referral	240 (13.5%)	204 (85.0%)	<0.001	5.725	(3.151–10.399)
Stroke education (patient)	1,005 (56.4%)	637 (63.4%)	<0.001	0.423	(0.288–0.622)
Understood FAST (patient)	328 (18.4%)	139 (42.4%)	0.002	0.48	(0.303–0.759)
Recognized the problem as a stroke (patient)	204 (11.5%)	44 (21.6%)	<0.001	0.165	(0.098–0.278)
Understand that stroke requires early treatment (family)	670 (37.6%)	259 (38.7%)	<0.001	0.542	(0.398–0.739)

*OR, odds ratio; CI, confidence interval*.

### Independent Factors Associated With Thrombolysis

During the study period, 267 patients arrived at the hospital within 4.5 h, and 103 (38.6%) received intravenous thrombolysis therapy. Multivariable logistic regression analysis identified that the following factors were independent predictive of thrombolysis receiving: under 65 years (OR, 3.585; 95% CI, 1.836–7.000), patient's high monthly income (3,000–5,000, OR, 3.935; 95% CI, 1.152–13.442; >5,000, OR, 15.995; 95% CI, 3.064–83.512), patient's high education level (6–12 years, OR, 3.410; 95% CI, 1.506–7.721. >12 years, OR, 7.685; 95% CI, 2.962–19.941), patient's ability to describe FAST (OR, 2.945; 95% CI, 1.407–6.164), family members understanding that stroke needs early treatment (OR, 3.466; 95% CI, 2.515–13.625), and the severity of stroke characterized by the NIHSS scores (7–15, OR: 27.222; 95% CI, 4.543–163.112. >15, OR: 16.654; 95% CI, 2.761–100.460). In contrast, late arrival was associate with a low thrombolysis rate (OR, 0.984; 95% CI, 0.977–0.991).

## Discussion

Thrombolysis is a time-dependent therapy and 1.9 million neuronal apoptosis could be avoided in the ischemic area per minute (15). Several factors affect the interval from stroke onset to hospital admission, but the influencing patterns and factors vary greatly across different populations and regions. We conducted this study to explore the factors that caused the prehospital delay and low rate of thrombolysis in AIS patients in China to enable more patients to arrive at the hospital within the time window. This study found that prehospital delay was severe in China, resulting a low thrombolysis rate. Only 15.0% of AIS patients arrived at the hospital within 4.5 h of stroke onset. The data was close to a previous study conducted in Chengdu, China (12), but lower than previous studies conducted in Eastern China and developed countries (8, 12, 16). The median prehospital delay time was 410 min, which was longer than that reported in north Spain (138.5 min) and Switzerland (150 min) (17), but shorter than that reported in the China QUEST (Quality Evaluation of Stroke Care and Treatment) study (15 h) (6). The thrombolysis rate in AIS patients was 5.8%, which was lower than the rates of studies in Jiangsu, China (18), London (12), and Nepal (19). The possible reason for this is that the health and education system lag behind due to its relatively backward economic development in Western China.

In this study, family members' knowledge about stroke and timely behavior was associated with the early arrival after stroke onset. Family members may play a great role in the use of Emergency Medical Service (EMS). There were 286 (16.0%) patients who arrived at the hospital by ambulance, among which 199 decision-makers were family members and 87 were patients themselves. The study in nonurban East Texas (USA) also showed that only 4.3% of 911 callers were AIS patients themselves and 60.1% were family members (20). Family member's discouragement to call 911 was the potential factor for the rarely EMS utilization (21, 22), and the behavior of waiting for family members was also the potential barrier to timely usage of EMS in the 40–74-year-old population (22). Previous studies also suggested that the bystander's prompt recognition, action, and emotional response were all associated with greater use of EMS and less delay (5, 9, 16, 20, 21, 23–26). However, few studies identified who made decisions on seeking medical help at the onset of a stroke (4, 22, 27), and no studies identified the role of cognitive and behavioral factors of the patient's family members in prehospital delay. This study further confirmed and extended the data: 41.5% of AIS patients' symptoms were first noticed by family members and 43.8% of medical help decisions were made by family members, similar to previous reports (4). The study showed that patients (instead of family members) deciding to seek medical services by themselves was independently associated with late arrival, which is similar to the results of a previous study (27). Family members' sufficient knowledge about timely stroke treatment was independently associated with early arrival and high thrombolysis rate, which could possibly result from cultural factors and family members' lack of stroke knowledge. We also found that living with descendants was associated with early arrival. Previous studies have identified that a majority of the elderly live with their descendants in Western and other regions of China (6–8), which is similar to the study in Korea (28) but higher than in other countries (9–11). The elderly often thought that the symptoms might disappear automatically and 24.2% of patients were afraid of troubling people that are not their family members, so living with their descendants was associated with early arrival. Furthermore, those who were over 65 years old were independently associated with late arrival and conservative therapy, which was consistent with previous studies results (6, 8, 14, 18, 29), but contrasted with others (11, 18, 30). Many people, especially the elderly, who suffer from neurological diseases may be less aware of the signs and symptoms of AIS compared to younger patients in Western China, and they were reluctant to seek medical attention unless they were advised to do so (26, 31). Therefore, to decrease the prehospital delay and the morbidity and mortality of AIS patients, public focus on family members is needed. In recent years, Stroke 1-2-0 Program provides a rapid recognition for stroke (32). It is suggested that more attention should be paid to the family members of stroke patients at high risk.

A majority of the statistically significant factors for the 4.5-h and for 6-h cutoff points were the same. This study demonstrated that patients who lived in non-urban areas and had a driving time from the onset place to the hospital of more than 30 min were independently associated with late arrival, which is similar to the results of previous studies (5, 17). Although the guidelines emphasized the importance of EMS (33), long driving time was still the main barrier to receiving AIS therapy due to the uneven distribution of hospitals and choosing to use private cars instead of using EMS in Western China (18). Patient's whose initial reaction was to call an ambulance, and arrived by ambulance (14, 16), were independently associated with early arrival. Despite previous studies showed that early arrival was not associated with patients' stroke knowledge (34), it is still believed that stroke education, the ability to describe FAST, and recognizing the problem as a stroke may shorten the prehospital delay (17, 28).

Similar to many other studies, the present study suggested that the factors such as severe stroke (12, 35–37), sudden onset symptoms (5, 34), onset at daylight (16, 19), consciousness disturbance (14, 36, 38, 39), weakness (38), and the use of an ambulance (5, 14, 18, 19, 28, 40) were significantly associated with early arrival. In comparison, those who had a prior stroke, a history of hyperlipidemia (18) or atrial fibrillation (5, 6, 18), had blurred vision in one or both eyes (38, 39) or were referred from other hospitals (6, 14, 41) were associated with delayed arrival. Among all the symptoms, only blurred vision was associated with prolonged prehospital delay, whereas weakness and consciousness disturbance could shorten the prehospital delay. Symptoms such as blurred vision, dizziness, and numbness are often believed by many patients to be relieved by lying down and having a rest. Therefore, milder symptoms should also be included in public stroke education for high-risk individuals.

It was also found that the decision time <60 min (40) was an independent predictor of early hospital arrival. During the study period, 180 patients decided to search for medical service within 30 min the symptoms occurred, among which 152 patients arrived at the hospital within 4.5 h while 28 patients did not. Among those who did not, most of them were live in suburban or rural with more than 1 h driving time, 13 patients were referred from other hospitals. A possible explanation for this might be the insufficient stroke knowledge and long distance. Some onset symptoms of a stroke are mild and similar to hypoglycemia, atrial fibrillation, or other diseases so they may visit the private clinic, community hospital or other departments instead of neurology or emergency departments. Previous studies have demonstrated that private clinics or other departments in different hospitals may differ from departments specialized in stroke recognition and treatment, so clinic doctors and community physicians should also be included in the target groups of the stroke (42).

A previous study confirmed that patients at any age could benefit from early thrombolysis (43). However, this study showed that the elderly was less likely to accept thrombolysis, which was similar to the study results in Singapore and the US (44). This may because the patients' family members worried more about the risk of bleeding from thrombolysis due to patients' underlying diseases and weaker systems. In contrast, people who have high monthly income, high education levels, high NIHSS scores were more likely to accept the thrombolysis therapy. For a poor family, the high cost of thrombolysis was the main barrier to accept the therapy (19, 45). In addition, cultural factors and economic status were also possible causes for this.

This study has several limitations. First, subjects' recall bias could occur inevitably, especially for symptom onset that could be accurate to within 10 min, and even within 30 min for some patients. Since reporting errors could go either way, their impact on our results cannot be determined. Second, patients' memory may be hampered by stroke-related cognitive impairment. Third, although every effort was made to ensure that subjects' interviews were conducted as soon as possible after hospitalization, interviews in some cases were delayed for various reasons. Lastly, missing data, including the participation refusal given by 8.1% of patients and the lack of 1.6% patients' information may have also adversely influenced the regression analyses.

## Conclusion

This is the first multicenter study to investigate the factors that influence the prehospital delay of thrombolysis for AIS, with a focus on the recognition and behavior of family members associated with prehospital delay in China. First, only 14.98% patients arrived at hospitals in Western China within 4.5 h, 40.52% patients arrived within 6 h after the onset of symptoms, 5.8% patients received thrombolysis. Second, the findings highlight the importance of the family member's recognition and behavior for early arrival. Third, the association between specific symptoms of stroke and prehospital delay were mixed so more studies should be carried out to further evaluate this link.

## Data Availability Statement

The original contributions presented in the study are included in the article/Supplementary Material, further inquiries can be directed to the corresponding author/s.

## Ethics Statement

The studies involving human participants were reviewed and approved by Research Ethics Committee of Chengdu Military General Hospital, Chengdu, China. The patients/participants provided their written informed consent to participate in this study.

## Author Contributions

RW contributed to the study design and original draft writing. ZW contributed to methodology and data curation. JW and CG performed data collection. YZ and LX conducted the statistical analysis. QW provided funding. DY performed supervision. All authors reviewed the draft and approved the final submitted version.

## Conflict of Interest

The authors declare that the research was conducted in the absence of any commercial or financial relationships that could be construed as a potential conflict of interest.
