# Supplementary material for: Early Hospital Arrival After Acute Ischemic Stroke Is Associated With Family Members' Knowledge About Stroke
Source: Front Neurol. 2021 May 26;12:652321. doi: 10.3389/fneur.2021.652321 (PMC8187751; doi:10.3389/fneur.2021.652321)
Supplement: Supplementary file 1 [file Table_1.DOCX]

**ONLINE SUPPLEMENT**

**Family Members’ Knowledge about Stroke Associated with Early Hospital Arrival after Acute Ischemic Stroke**

| Supplemental Table I. Patient Characteristics, Time to Presentation | | | | | | |
| --- | --- | --- | --- | --- | --- | --- |
| Factors | | Total (%) | ≥4.5 hours (%) | *P* | ≥6 hours (%) | *P* |
| Overall rate (N, %) | | 1782 (100%) | 1515 (85.0%) | | 1060 (59.5%) | |
| Gender: | |  |  | 0.403 |  | 0.262 |
|  | Male | 1033 (58.0%) | 872 (84.4%) |  | 603 (58.4%) |  |
|  | Female | 749 (42.0%) | 643 (85.8%) |  | 457 (61.0%) |  |
| Age: | |  |  | <0.001 |  | <0.001 |
|  | <65 years | 596 (33.4%) | 462 (77.5%) |  | 304 (51.0%) |  |
|  | ≥65 years | 1186 (66.6%) | 1053 (88.8%) | | 756 (63.7%) |  |
| Marital status: | |  |  | 0.406 |  | 0.896 |
|  | Married | 1575 (88.4%) | 1335 (84.8%) | | 936 (59.4%) |  |
|  | Unmarried | 207 (11.6%) | 180 (87.0%) |  | 124 (60.0%) |  |
| Lives: | |  |  | 0.027 |  | <0.001 |
|  | Alone | 71 (4.0%) | 68 (95.8%) |  | 66 (93.0%) |  |
|  | With descendants | 1692 (95.0%) | 1432 (84.6%) | | 981 (58.0%) |  |
|  | Senile apartment | 19 (1.1%) | 15 (79.0%) |  | 13 (68.4%) |  |
| Area resides in: | |  |  | <0.001 |  | <0.001 |
|  | Urban | 928 (52.1%) | 704 (75.9%) |  | 404 (43.5%) |  |
|  | Suburban | 577 (32.4%) | 552 (95.7%) |  | 426 (73.8%) |  |
|  | Rural | 277 (15.5%) | 259 (93.5%) |  | 230 (83.0%) |  |
| Driving time to hospital (min): | | |  | <0.001 |  | <0.001 |
|  | <30 min | 327 (18.4%) | 246 (75.2%) |  | 155 (47.4%) |  |
|  | 30-60 min | 493 (27.7%) | 394 (79.9%) |  | 272 (55.2%) |  |
|  | >60 min | 962 (54.0%) | 875 (91.0%) |  | 633 (65.8%) |  |
| Monthly income of patient (yuan^*^): | | | | 0.833 |  | 0.047 |
|  | <1000 | 138 (7.7%) | 116 (84.1%) |  | 84 (60.9%) |  |
|  | 1000-3000 | 836 (46.9%) | 714 (85.4%) |  | 504 (60.3%) |  |
|  | 3000-5000 | 695 (39.0%) | 592 (85.2%) |  | 419 (60.3%) |  |
|  | >5000 | 113 (6.3%) | 93 (82.3%) |  | 53 (46.9%) |  |
| Medical insurance: | |  |  | 0.82 |  | 0.811 |
|  | YES | 1713 (96.1%) | 1457 (85.1%) | | 1018 (59.4%) | |
|  | NO | 69 (3.9%) | 58 (84.1%) |  | 42 (60.9%) |  |
| Education level of patient: | | |  | 0.022 |  | <0.001 |
|  | <6 years | 581 (32.6%) | 506 (87.1%) |  | 384 (66.1%) |  |
|  | 6y-12 years | 864 (48.5%) | 738 (85.4%) |  | 491 (56.8%) |  |
|  | >12 years | 337 (18.9%) | 271 (80.4%) |  | 185 (54.9%) |  |
| Prior stroke: | |  |  | <0.001 |  | <0.001 |
|  | YES | 397 (22.3%) | 376 (94.7%) |  | 322 (53.3%) |  |
|  | NO | 1385 (77.7%) | 1139 (82.2%) | | 738 (53.3%) |  |
| History of hypertension: | | |  | <0.001 |  | <0.001 |
|  | YES | 479 (26.9%) | 376 (78.5%) |  | 248 (51.8%) |  |
|  | NO | 1303 (73.1%) | 1139 (87.4%) | | 812 (62.3%) |  |
| History of diabetes: | | |  | 0.601 |  | 0.135 |
|  | YES | 392 (22.0%) | 330 (84.2%) |  | 246 (62.8%) |  |
|  | NO | 1390 (78.0%) | 1185 (85.3%) | | 814 (58.6%) |  |
| History of hyperlipidemia: | | |  | 0.83 |  | 0.076 |
|  | NO | 1549 (86.9%) | 1318 (85.1%) | | 909 (58.7%) |  |
|  | YES | 233 (13.1%) | 197 (84.6%) |  | 151 (64.8%) |  |
| History of atrial fibrillation: | | |  | 0.002 |  | <0.001 |
|  | YES | 134 (7.5%) | 126 (94.0%) |  | 100 (74.6%) |  |
|  | NO | 1648 (92.5%) | 1389 (84.3%) | | 960 (58.3%) |  |
| Smoker: | |  |  | 0.053 |  | 0.065 |
|  | YES | 466 (26.2%) | 409 (87.8%) |  | 294 (63.1%) |  |
|  | NO | 1316 (73.8%) | 1106 (84.0%) | | 766 (58.2%) |  |
| Onset time: | |  |  | <0.001 |  | <0.001 |
|  | Daytime | 1488 (83.5%) | 1245 (83.7%) | | 832 (55.9%) |  |
|  | Nighttime | 294 (16.5%) | 270 (91.8%) |  | 228 (77.6%) |  |
| Onset location: | |  |  | 0.001 |  | 0.001 |
|  | Home | 1467 (82.3%) | 1267 (86.4%) | | 898 (61.2%) |  |
|  | Other | 315 (17.7%) | 248 (78.7%) |  | 162 (51.4%) |  |
| Symptom onset: | |  |  | <0.001 |  | <0.001 |
|  | Gradual | 495 (27.8%) | 476 (96.2%) |  | 363 (73.3%) |  |
|  | Sudden | 1287 (72.2%) | 1039 (80.7%) | | 697 (54.2%) |  |
| NIHSS score: | |  |  | <0.001 |  | <0.001 |
|  | <7 | 517 (29.0%) | 501 (96.9%) |  | 365 (70.6%) |  |
|  | 7-15 | 946 (53.1%) | 776 (82.0%) |  | 527 (55.7%) |  |
|  | >15 | 319 (17.9%) | 238 (74.6%) |  | 168 (52.7%) |  |
| Loss of consciousness: | | |  | <0.001 |  | <0.001 |
|  | YES | 110 (6.2%) | 57 (51.8%) |  | 25 (22.7%) |  |
|  | NO | 1672 (93.8%) | 1458 (87.2%) | | 1035 (61.9%) | |
| Weakness: | |  |  | <0.001 |  | <0.001 |
|  | YES | 1299 (72.9%) | 1046 (80.5%) | | 728 (56.0%) |  |
|  | NO | 483 (27.1%) | 469 (97.1%) |  | 332 (68.7%) |  |
| Numbness: | |  |  | <0.001 |  | 0.001 |
|  | YES | 1068 (59.9%) | 852 (79.8%) |  | 600 (56.2%) |  |
|  | NO | 714 (40.1%) | 663 (92.9%) |  | 460 (64.4%) |  |
| Gait abnormality: | |  |  | <0.001 |  | <0.001 |
|  | YES | 663 (37.2%) | 514 (77.5%) |  | 345 (52.0%) |  |
|  | NO | 1119 (62.8%) | 1001 (89.5%) | | 715 (63.9%) |  |
| Difficulty speaking: | | |  | <0.001 |  | 0.001 |
|  | YES | 552 (31.0%) | 447 (81.0%) |  | 297 (53.8%) |  |
|  | NO | 1230 (69.0%) | 1068 (86.8%) | | 763 (62.0%) |  |
| Blurred vision: | |  |  | <0.001 |  | 0.003 |
|  | YES | 523 (29.4%) | 415 (79.3%) |  | 283 (54.1%) |  |
|  | NO | 1259 (70.7%) | 1100 (87.4%) | | 777 (61.7%) |  |
| Dizziness: | |  |  | 0.017 |  | 0.149 |
|  | YES | 258 (14.5%) | 232 (89.9%) |  | 164 (63.6%) |  |
|  | NO | 1524 (85.5%) | 1283 (84.2%) | | 896 (58.8%) |  |
| First noticed symptoms: | | |  | <0.001 |  | <0.001 |
|  | Patient | 1029 (57.74%) | 939 (91.3%) |  | 691 (67.2%) |  |
|  | Family member | 739 (41.5%) | 566 (77.0%) |  | 362 (49.0%) |  |
|  | Others | 14 (0.8%) | 10 (71.4%) |  | 7 (50.0%) |  |
| Patient’s initial reaction: | | |  | <0.001 |  | <0.001 |
|  | Called ambulance | 390 (21.9%) | 229 (58.7%) |  | 91 (23.3%) |  |
|  | Went directly to hospital | 591 (33.2%) | 562 (95.1%) |  | 426 (72.1%) |  |
|  | Contacted relative | 465 (26.1%) | 420 (90.3%) |  | 321 (69.0%) |  |
|  | Waited | 336 (18.9%) | 304 (90.5%) |  | 222 (66.1%) |  |
| Attribution to other reasons: | | |  | <0.001 |  | <0.001 |
|  | YES | 364 (20.4%) | 351 (96.4%) |  | 249 (68.4%) |  |
|  | NO | 1418 (79.6%) | 1164 (82.1%) | | 811 (57.2%) |  |
| Concern about troubling others: | | |  | <0.001 |  | <0.001 |
|  | YES | 431 (24.2%) | 418 (97.0%) |  | 327 (75.9%) |  |
|  | NO | 1351 (75.8%) | 1097 (81.2%) | | 733 (54.3%) |  |
| Arrival through referral: | | |  | <0.001 |  | <0.001 |
|  | YES | 240 (13.5%) | 227 (94.6%) |  | 204 (85.0%) |  |
|  | NO | 1542 (86.5%) | 1288 (83.5%) | | 856 (55.5%) |  |
| Arrival by ambulance: | | |  | <0.001 |  | <0.001 |
|  | YES | 286 (16.0%) | 95 (33.2%) |  | 26 (9.1%) |  |
|  | NO | 1496 (84.0%) | 1420 (94.9%) | | 1034 (69.1%) | |
| Stroke education (patient): | | |  | <0.001 |  | <0.001 |
|  | YES | 1005 (56.4%) | 885 (88.1%) |  | 637 (63.4%) |  |
|  | NO | 777 (43.6%) | 630 (81.1%) |  | 423 (54.4%) |  |
| Able to describe FAST (patient): | | |  | <0.001 |  | <0.001 |
|  | YES | 328 (18.4%) | 257 (78.4%) |  | 139 (42.4%) |  |
|  | NO | 1454 (81.6%) | 1258 (86.5%) | | 921 (63.3%) |  |
| Recognized the problem as a stroke (patient): | | | | <0.001 |  | <0.001 |
|  | YES | 204 (11.5%) | 145 (71.1%) |  | 44 (21.6%) |  |
|  | NO | 1578 (88.6%) | 1370 (86.8%) | | 1016 (64.4%) | |
| Understood that stroke requires early treatment (patient): | | | | <0.001 |  | <0.001 |
|  | YES | 354 (19.9%) | 272 (76.8%) |  | 150 (42.4%) |  |
|  | NO | 1428 (80.1%) | 1243 (87.0%) | | 910 (63.7%) |  |
| Understood time window of stroke treatment (patient): | | | | 0.265 |  | <0.001 |
|  | YES | 137 (7.7%) | 112 (81.8%) |  | 47 (34.3%) |  |
|  | NO | 1645 (92.3%) | 1403 (85.3%) | | 1013 (61.6%) | |
| Individual seeking medical services/decision maker: | | | | <0.001 |  | <0.001 |
|  | Family member | 781 (43.8%) | 603 (77.2%) |  | 387 (49.6%) |  |
|  | Patient | 1001 (56.2%) | 912 (91.1%) |  | 673 (67.2%) |  |
| Decision time: | |  |  | <0.001 |  | <0.001 |
|  | <30 min | 180 (10.1%) | 28 (15.6%) |  | 8 (4.4%) |  |
|  | 30-60 min | 164 (9.2%) | 63 (38.4%) |  | 6 (3.7%) |  |
|  | >60 min | 1438 (80.7%) | 1424 (99.0%) | | 1046 (72.7%) | |
| Monthly income (yuan*,family member): | | |  | <0.001 |  | <0.001 |
|  | 1000< | 9 (0.51%) | 7 (77.8%) |  | 5 (55.6%) |  |
|  | 1000-3000 | 610 (34.2%) | 554 (90.8%) |  | 414 (67.9%) |  |
|  | 3000-5000 | 608 (34.1%) | 498 (81.9%) |  | 329 (54.1%) |  |
|  | >5000 | 555 (31.1%) | 456 (82.2%) |  | 312 (56.2%) |  |
| Education (family member): | | |  | <0.001 |  | <0.001 |
|  | <6 years | 263 (14.8%) | 256 (97.3%) |  | 204 (77.6%) |  |
|  | 6y-12 years | 902 (50.6%) | 817 (90.6%) |  | 545 (60.4%) |  |
|  | >12 years | 617 (34.6%) | 442 (71.6%) |  | 311 (50.4%) |  |
| Stroke education (family member): | | |  | <0.001 |  | <0.001 |
|  | YES | 1091 (61.2%) | 847 (77.6%) |  | 546 (50.1%) |  |
|  | NO | 691 (38.8%) | 668 (96.7%) |  | 514 (74.4%) |  |
| Able to describe FAST (family member): | | | | <0.001 |  | <0.001 |
|  | YES | 642 (36.0%) | 445 (69.3%) |  | 259 (40.3%) |  |
|  | NO | 1140 (64.0%) | 1070 (93.9%) | | 801 (70.3%) |  |
| Recognized the problem as stroke (family member): | | | | <0.001 |  | <0.001 |
|  | YES | 586 (32.9%) | 382 (65.2%) |  | 225 (38.4%) |  |
|  | NO | 1196 (67.1%) | 1133 (94.7%) | | 835 (69.8%) |  |
| Understood stroke requires early treatment (family member): | | | | <0.001 |  | <0.001 |
|  | YES | 670 (37.6%) | 462 (69.0%) |  | 259 (38.7%) |  |
|  | NO | 1111 (62.3%) | 1052 (94.7%) | | 801 (72.1%) |  |
| Understood time window of stroke treatment (family member): | | | | <0.001 |  | <0.001 |
|  | YES | 252 (14.1%) | 188 (74.6%) |  | 103 (40.9%) |  |
|  | NO | 1530 (85.9%) | 1327 (86.7%) | | 957 (62.6%) |  |

NIHSS: National Institutes of Health Stroke Scale; FAST: Face, Arm, Speech, Time.
*1000 yuan = 153.25 dollar.
